# Association of glycated hemoglobin A_1c_ levels with cardiovascular outcomes in the general population: results from the BiomarCaRE (Biomarker for Cardiovascular Risk Assessment in Europe) consortium

**DOI:** 10.1186/s12933-021-01413-4

**Published:** 2021-11-15

**Authors:** Christoph Sinning, Nataliya Makarova, Henry Völzke, Renate B. Schnabel, Francisco Ojeda, Marcus Dörr, Stephan B. Felix, Wolfgang Koenig, Annette Peters, Wolfgang Rathmann, Ben Schöttker, Hermann Brenner, Giovanni Veronesi, Giancarlo Cesana, Paolo Brambilla, Tarja Palosaari, Kari Kuulasmaa, Inger Njølstad, Ellisiv Bøgeberg Mathiesen, Tom Wilsgaard, Stefan Blankenberg, Stefan Söderberg, Marco M. Ferrario, Barbara Thorand

**Affiliations:** 1 Department of Cardiology, University Heart & Vascular Center Hamburg, Martinistr. 52, 20246 Hamburg, Germany; 2 German Center for Cardiovascular Research (DZHK), Partner Site Hamburg/Kiel/Lübeck, Hamburg, Germany; 3 Department of Study of Health in Pomerania/Clinical-Epidemiological Research, Institute for Community Medicine, University Medicine Greifswald, Greifswald, Germany; 4 German Center for Cardiovascular Research (DZHK), Partner Site Greifswald, Greifswald, Germany; 5 Department of Internal Medicine B, University of Medicine Greifswald, Greifswald, Germany; 6 German Heart Center Munich, Technical University, Munich, Germany; 7 German Center for Cardiovascular Research (DZHK), Partner Site Munich Heart Alliance, Munich, Germany; 8 Institute of Epidemiology and Medical Biometry, University of Ulm, Ulm, Germany; 9 German Research Center for Environmental Health, Institute of Epidemiology, Helmholtz Zentrum München, Neuherberg, Germany; 10 Institute for Biometrics and Epidemiology, German Diabetes Center, Leibniz Institute for Diabetes Research at Heinrich Heine University, Düsseldorf, Germany; 11 Division of Clinical Epidemiology and Ageing Research, German Cancer Research Center, Heidelberg, Germany; 12 Network Aging Research, University of Heidelberg, Heidelberg, Germany; 13 Department of Medicine and Surgery, EPIMED Research Center, University of Insubria at Varese, Varese, Italy; 14 Department of Medicine and Surgery, University of Milano-Bicocca, Milan, Italy; 15 Finnish Institute for Health and Welfare, Division Public Health and Welfare, Helsinki, Finland; 16 Department of Community Medicine, UiT The Arctic University of Norway, Tromsö, Norway; 17 Brain and Circulation Research Group, UiT The Arctic University of Norway, Tromsö, Norway; 18 Neurological Department, University Hospital of North Norway, Tromsö, Norway; 19 Department of Public Health and Clinical Medicine, Umeå University, Umeå, Sweden; 20 German Center for Diabetes Research (DZD), Munich, Neuherberg, Germany; 21 Institute for Health Services Research in Dermatology and Nursing (IVDP), University Medical Center Hamburg-Eppendorf, Hamburg, Germany

**Keywords:** Biomarkers, Glycated hemoglobin A_1c_ (HbA_1c_), Cardiovascular risk, Mortality, BiomarCaRE (Biomarker for Cardiovascular Risk Assessment in Europe), MORGAM (MONICA Risk Genetics Archiving and Monograph)

## Abstract

**Background:**

Biomarkers may contribute to improved cardiovascular risk estimation. Glycated hemoglobin A_1c_ (HbA_1c_) is used to monitor the quality of diabetes treatment. Its strength of association with cardiovascular outcomes in the general population remains uncertain. This study aims to assess the association of HbA_1c_ with cardiovascular outcomes in the general population.

**Methods:**

Data from six prospective population-based cohort studies across Europe comprising 36,180 participants were analyzed. HbA_1c_ was evaluated in conjunction with classical cardiovascular risk factors (CVRFs) for association with cardiovascular mortality, cardiovascular disease (CVD) incidence, and overall mortality in subjects without diabetes (N = 32,496) and with diabetes (N = 3684).

**Results:**

Kaplan–Meier curves showed higher event rates with increasing HbA_1c_ levels (log-rank-test: p < 0.001). Cox regression analysis revealed significant associations between HbA_1c_ (in mmol/mol) in the total study population and the examined outcomes. Thus, a hazard ratio (HR) of 1.16 (95% confidence interval (CI) 1.02–1.31, p = 0.02) for cardiovascular mortality, 1.13 (95% CI 1.03–1.24, p = 0.01) for CVD incidence, and 1.09 (95% CI 1.02–1.17, p = 0.01) for overall mortality was observed per 10 mmol/mol increase in HbA_1c_. The association with CVD incidence and overall mortality was also observed in study participants without diabetes with increased HbA_1c_ levels (HR 1.12; 95% CI 1.01–1.25, p = 0.04) and HR 1.10; 95% CI 1.01–1.20, p = 0.02) respectively. HbA_1c_ cut-off values of 39.9 mmol/mol (5.8%), 36.6 mmol/mol (5.5%), and 38.8 mmol/mol (5.7%) for cardiovascular mortality, CVD incidence, and overall mortality, showed also an increased risk.

**Conclusions:**

HbA_1c_ is independently associated with cardiovascular mortality, overall mortality and cardiovascular disease in the general European population. A mostly monotonically increasing relationship was observed between HbA_1c_ levels and outcomes. Elevated HbA_1c_ levels were associated with cardiovascular disease incidence and overall mortality in participants without diabetes underlining the importance of HbA_1c_ levels in the overall population.

**Supplementary Information:**

The online version contains supplementary material available at 10.1186/s12933-021-01413-4.

## Introduction

Assessing the cardiovascular risk in the general population is important for clinical decision-making, including the prescription of medication or targeting of lifestyle intervention strategies [1–3]. Despite the identification of novel independent biomarkers, assessment of cardiovascular risk relies on a set of traditional cardiovascular risk factors (CVRFs) such as age, sex, blood pressure, lipid levels, diabetes mellitus (DM), and smoking. The decision whether to include novel biomarkers in cardiovascular risk assessment remains a topic of intense debate and research [4]. Diabetes is regarded as a classical risk factor for cardiovascular disease (CVD) [5]. Due to the clinical need to identify novel risk factors to improve cardiovascular risk assessment, glycated hemoglobin or hemoglobin A_1c_ (HbA_1c_) may be a potential candidate [6]. The primary clinical use of HbA_1c_ is as an indicator of the average blood glucose levels over the past 3 months and thus it is used as a diagnostic and screening tool for DM [7, 8].

While associations between HbA_1c_ levels and the risk of cardiovascular outcomes or overall mortality have been reported [9–14], only few studies suggested that HbA_1c_ may be associated with cardiovascular outcomes in an apparently healthy population [9, 15–19]. Recently published results underlined the additional use of HbA_1c_ levels in middle-aged individuals without a history of CVD and HbA_1c_ levels in the nondiabetic range [1]. Studies employing Mendelian randomization support a link between increasing HbA_1c_ levels and an increased risk of coronary artery disease [20, 21]. In this context, HbA_1c_ might be associated with an increased risk and could be of importance in individuals without a diagnosis of diabetes [4].

In the present study, we evaluated the distribution of HbA_1c_ levels in population-based cohorts across Europe. Furthermore, we analyzed the association of continuous HbA_1c_ levels with cardiovascular mortality, CVD incidence, and overall mortality. In addition, the association between HbA_1c_ levels and time-to-event was analyzed in subgroups with and without diabetes and according to age. Finally, cut-offs for the dichotomization of HbA_1c_ were determined for each outcome.

## Methods

### Study overview

The design and rationale of the Biomarker for Cardiovascular Risk Assessment across Europe (BiomarCaRE) project have been described previously [22]. Briefly, BiomarCaRE is based on the MORGAM (MONICA Risk Genetics Archiving and Monograph) Project. The MORGAM/BiomarCaRE Data Center in Helsinki harmonized individual data from 21 population-based cohort studies with central storage of selected biomaterial of more than 300,000 participants [22]. Using the harmonized database of the BiomarCaRE project (FP7/2007–2013) [22], we analyzed individual data of 36,180 study participants with available HbA_1c_ levels.

### Study cohorts

The present analysis included six cohort studies from four European countries (Germany, Italy, Sweden, and Norway), namely the Cooperative Health Research in the Region of Augsburg (KORA) Study, the Study of Health in Pomerania (SHIP), and the Epidemiologische Studie zu Chancen der Verhütung, Früherkennung und optimierten THerapie chronischer ERkrankungen in der älteren Bevölkerung (ESTHER) Study, all from Germany, the MONICA Brianza Study from Italy, the Northern Sweden MONICA Study from Sweden, and the Tromsø Study from Norway. Each cohort is based on a well-defined population (see Additional file 1: Table S1).

For each cohort, the following harmonized variables were available at baseline: duration of time with diagnosed diabetes in years, age, sex, smoking status, body-mass-index (BMI), systolic blood pressure, diastolic blood pressure, total cholesterol, high-density lipoprotein (HDL) cholesterol, HbA_1c_, and history of DM. The history of DM was defined as documented or self-reported history of diabetes. This variable includes both type 1 and type 2 DM. Detailed definitions of this variable in each cohort are provided in Additional file 1: Table S1. Also participants not diagnosed with DM but with high HbA_1c_ levels (> 48 mmol/mol/6.5%—a diagnostic criterion for clinical DM) were assigned to the DM group. Additional sub-classification into type 1 or type 2 DM was not possible with this dataset. Treatment of DM was available as (a) medication with insulin, (b) oral medication, but no insulin and (c) diet only.

Smoking status was determined based on self-reports. BMI, systolic blood pressure, diastolic blood pressure, total cholesterol, and HDL cholesterol parameters were measured (high blood pressure was defined either as systolic blood pressure ≥ 140 mmHg and/or diastolic blood pressure ≥ 90 mmHg) and it was recorded whether the patient was on antihypertensive medication. Prevalent CVD, like previous myocardial infarction or stroke, was assessed using the documented or self-reported history of myocardial infarction or stroke, including angina pectoris when the data did not permit its distinction from myocardial infarction. In MORGAM, prevalent heart failure was assessed with the item documented or self-reported history of heart failure.

### Study outcome

The following outcome measures were defined: (I) cardiovascular mortality, (II) CVD incidence, and (III) overall mortality, defined as mortality due to any cause during follow-up. Follow-up commenced at the baseline examination date [23].

Cardiovascular mortality included death due to coronary heart disease or stroke. Cardiovascular disease, as an endpoint, was defined as the first fatal or non-fatal coronary event or possible ischemic stroke. Coronary events included acute definite or possible myocardial infarction or coronary death, unstable angina pectoris, cardiac revascularization, and unclassifiable death (i.e., death with insufficient evidence of coronary origin and no competing cause). In the MONICA/KORA Augsburg study, cardiac revascularization was not followed-up. In the MONICA/KORA Augsburg and MONICA Brianza studies, unstable angina pectoris was not assessed as an outcome but primarily included in the category “possible myocardial infarction” of the WHO MONICA classification used in these studies.

### Laboratory procedures

HbA_1c_ was measured using whole blood. All HbA_1c_ measurements were performed upon study entry to avoid glycation of blood samples during storage (except the Northern Sweden MONICA cohort measuring HbA_1c_ levels in samples that had been stored at − 80 °C). Locally measured HbA_1c_ values were transferred directly to the MORGAM Data Center (except for the SHIP study). The assays either reported their results as percentages (%), following the National Glycohemoglobin Standardization Program (NGSP), or in units of mmol/mol if they had employed the International Federation of Clinical Chemistry (IFCC) consensus reference method. Data from cohorts which reported their values as percentages were converted to mmol/mol using the standard formula: IFCC = 10.93 * NGSP − 23.50.

### Statistical analyses

Unadjusted and age-adjusted Kaplan–Meier survival curves for cardiovascular mortality, CVD incidence, and overall mortality were computed based on HbA_1c_ tertiles. For the age-adjusted Kaplan–Meier survival analyses the three HbA_1c_ tertiles were < 34.4 mmol/mol (5.3%), 34.4 mmol/mol (5.3%) − 38.8 mmol/mol (5.7%); and > 38.8 mmol/mol (5.7%). To adjust the survival curves for the age distribution in the data, the following procedures were applied: (a) age was categorized using cut-offs 35, 40, 45, 50, 55, 60, 65, and 70 years; (b) an individual belonging to a particular HbA_1c_ tertile and to age category j was assigned a weight equal to (n_j_/N)/(n_ij_/N), where N is the total sample size, n_j_ is the number of individuals in age category j, and n_ij_ is the number of individuals in age category j belonging to the HbA_1c_ tertile; (c) these weights were then applied to the observations for estimating the Kaplan–Meier curves. A log-rank test was used to compare the unadjusted survival curves. The adjusted survival curves we compared using a robust score test obtained from a weighted Cox model with HbA_1c_ categorized using tertiles as the only predictor. Follow-up time quartiles were estimated by the Kaplan–Meier potential follow-up estimator [24].

Sex- and cohort-stratified Cox proportional hazards models for cardiovascular mortality, CVD incidence, and overall mortality were computed using individual-level data from the available cohorts. For these analyses the HbA_1c_ (in mmol/mol) was applied untransformed and used as continuous variable. The Cox models for the three endpoints were adjusted for age (time scale), sex and cohort (strata), and CVRFs, smoking status (daily smoker yes/no), BMI, systolic blood pressure, DM (yes/no), total cholesterol to HDL cholesterol ratio and treatment of DM. Two separate extensions of the models were considered. In one extension, baseline age groups < 55, 55–64, ≥ 65 years at baseline and their interaction with continuous untransformed HbA_1c_ was added to the models. In the second extension, an interaction between continuous untransformed HbA_1c_ and the group indicator for diabetes was added. For these models results the HbA_1c_ hazard ratios are presented in forest plots and tables. In the former HbA_1c_ hazard ratios are presented per 10 mmol/mol increase and in the later per 1 mmol/mol increase (Additional file 1). To assess the linearity assumption of HbA_1c_ used in the previous Cox regressions, additional Cox models with HbA_1c_ were formulated using penalized cubic splines. C-indices were computed using these last models and compared against a model including all other predictors with the exception of HbA_1c_. Further details are given in Additional file 1. HbA_1c_ cut-offs that intend to separate subjects into low- and high-risk groups were calculated for each endpoint using the method of Contal and O’Quigley [25]. This method examines a rescaled version of the log-rank test statistic for each possible cut-off and selects the cut-off that maximizes the rescaled log-rank test statistic. Equality of survival curves in the groups separated by the optimal cut-off values was tested using the methods described by Contal and O’Quigley [25].

Individuals with CVD at baseline were excluded in the survival analyses using CVD as endpoint. There were no exclusions based on prevalent disease for the two other endpoints.

A two-sided P-value of < 0.05 was considered statistically significant. Adjustment for multiple testing was not performed due to the exploratory nature of the analyses [26]. All statistical methods were implemented in R statistical software version 4.1.1 (http://www.R-project.org) [27].

## Results

### Baseline characteristics

The baseline characteristics for individuals with available HbA_1c_ measurements for the entire cohort and for individuals with and without DM are shown in Table 1. The characteristics of each cohort are summarized in Additional file 1: Table S2.

**Table 1:** Baseline characteristics of the entire study population

	All (N = 36,180)	No diabetes (N = 32,496)	Prevalent diabetes (N = 3684)
Baseline characteristics			
Survey year	1987–2012	1987–2012	1987–2012
Examination age (years)	57.4 (47.0, 65.1)	56.4 (45.3, 64.5)	64.0 (57.9, 69.0)
Male (%)	17,069 (47.2)	15,095 (46.5)	1974 (53.6)
BMI (kg/m^2^)	26.4 (23.8, 29.4)	26.1 (23.6, 29.0)	29.2 (26.4, 32.6)
Daily smoker (%)	8243 (27.7)	7602 (27.9)	641 (25.7)
Hypertension (%)	17,084 (47.5)	14,506 (44.9)	2578 (70.6)
Systolic BP (mmHg)	133.5 (120.0, 149.0)	132.0 (120.0, 147.0)	140.0 (130.0, 155.5)
Diastolic BP (mmHg)	80.0 (74.0, 90.0)	80.0 (74.0, 89.5)	80.5 (76.0, 90.0)
Antihypertensive (%)	7827 (21.7)	6057 (18.7)	1770 (48.5)
Diabetes (%)	3684 (10.2)	0 (0)	3684 (100)
Diabetes treatment: none (%)	33,834 (95.1)	32,496 (100)	1338 (43.5)
Diabetes treatment: insulin (%)	598 (1.7)	0 (0)	598 (19.4)
Diabetes treatment: tablets, but no insulin (%)	1007 (2.8)	0 (0)	1007 (32.7)
Diabetes treatment: dietary (%)	133 (0.4)	0 (0)	133 (4.3)
Family history of CHD (%)	4716 (18.6)	4242 (18.7)	474 (17.6)
Prev. MI or stroke (%)	2132 (6.0)	1587 (4.9)	545 (15.3)
History of MI (%)	1417 (4.0)	1060 (3.3)	357 (10.0)
Prev. stroke (%)	862 (2.4)	624 (1.9)	238 (6.6)
History of heart failure (%)	1454 (5.7)	1046 (4.7)	408 (13.2)
Endpoints			
Cardiovascular mortality (%)	1392 (3.9)	1080 (3.3)	312 (8.5)
Cardiovascular disease (%)	2339 (8.2)	2043 (7.8)	296 (12.1)
Overall mortality (%)	4601 (12.7)	3768 (11.6)	833 (22.7)
Biomarkers			
HbA_1c_ (mmol/mol)	36.6 (32.2, 39.9)	35.5 (32.2, 38.8)	50.8 (44.3, 59.6)
HbA_1c_ (%)	5.5 (5.1, 5.8)	5.4 (5.1, 5.7)	6.8 (6.2, 7.6)
Total cholesterol (mmol/L)	5.9 (5.0, 6.7)	5.9 (5.1, 6.8)	5.7 (4.8, 6.5)
HDL cholesterol (mmol/L)	1.4 (1.2, 1.7)	1.4 (1.2, 1.7)	1.2 (1.0, 1.4)

Baseline characteristics are presented as absolute and relative frequencies for categorical variables, and quartiles (medians with 25th and 75th percentiles) for continuous variables as well as range in years for years of baseline examinations

The numbers provided for the cardiovascular disease endpoint are after excluding those individuals with history of cardiovascular disease

*BMI* body mass index, *BP* blood pressure, *CHD* coronary heart disease, *HDL* high density lipoprotein, *LDL* low density lipoprotein, *MI* myocardial infarction

For 36,180 subjects, HbA_1c_ measurements and information regarding diabetes status were available. Men and women were represented almost equally (19,111 women; 52.8%). The median age was 57.4 years, the median BMI 26.4 kg/m^2^, and the median systolic blood pressure 133.5 mmHg. At baseline, approximately 28% of the study cohort were daily smokers, 47.5% had high blood pressure or were taking antihypertensive medication, and 10.2% had a diagnosis of diabetes.

### Distribution of HbA_1c_ levels in the cohort

The distribution of HbA_1c_ levels in the entire cohort and each cohort study are shown in Additional file 1: Fig. S1. The median HbA_1c_ was 36.6 mmol/mol (5.5%). The 25th and 75th percentiles were 32.2 mmol/mol (5.1%) and 39.9 mmol/mol (5.8%), respectively.

### HbA_1c_ levels and association with cardiovascular mortality, cardiovascular disease, and overall mortality

The maximum follow-up time was 21.9 years. During the median follow-up time of 9.9 years, 1392 cases of cardiovascular death, 2711 cases of CVD, and 4,601 deaths due to any cause were observed. Further information on the median follow-up for each cohort is provided in Additional file 1: Table S3.

**Fig. 1:**
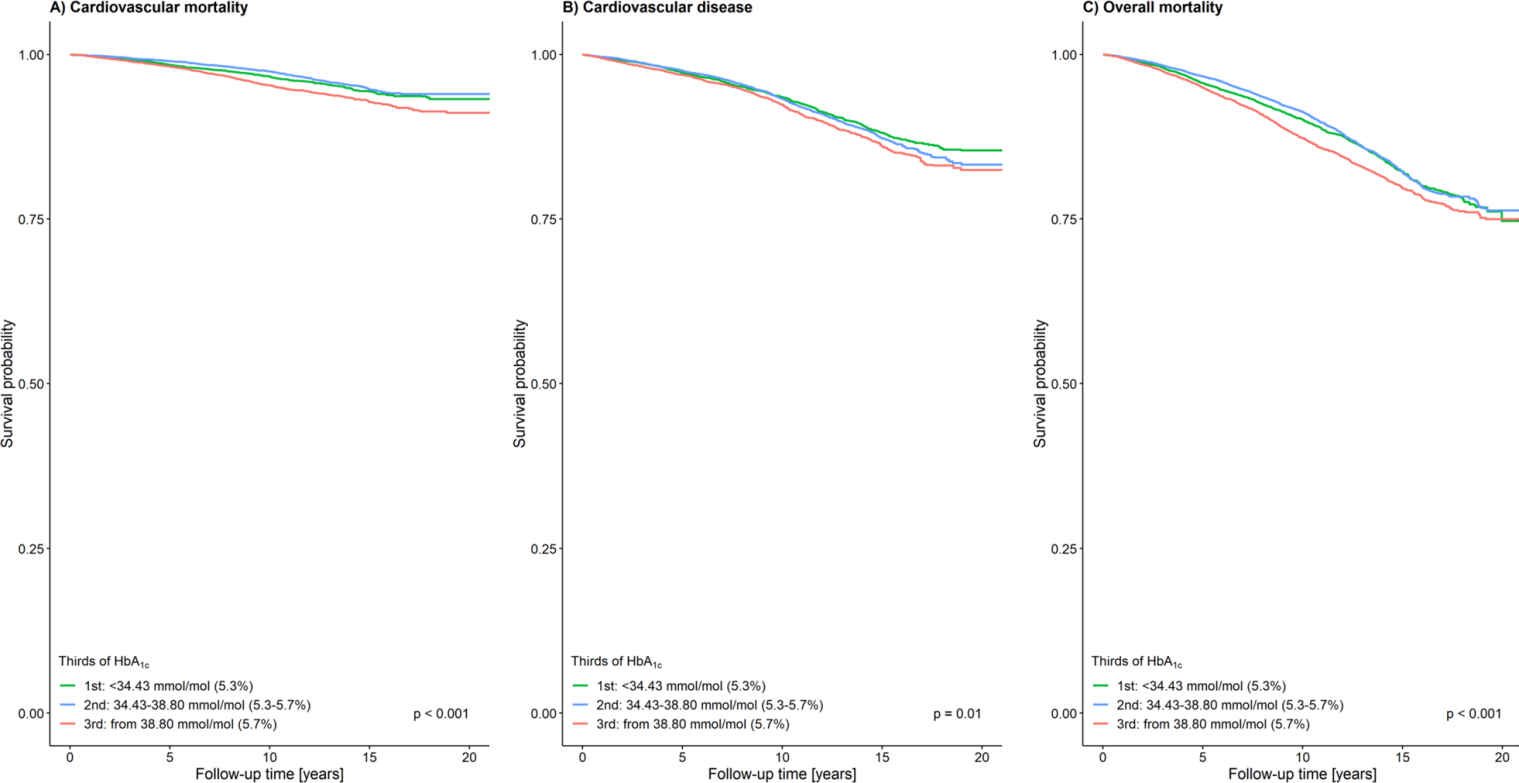
Age-adjusted Kaplan–Meier curves of **A** cardiovascular mortality, **B** cardiovascular disease, and **C** overall mortality for each HbA_1c_ tertile

As illustrated in the age-adjusted Kaplan–Meier survival analyses for the three HbA_1c_ tertiles < 34.4 mmol/mol (5.3%), 34.4 mmol/mol (5.3%) − 38.8 mmol/mol (5.7%); and > 38.8 mmol/mol (5.7%) the probability of all investigated outcomes increased with increasing HbA_1c_ levels (Additional file 1: Fig. S2). Adjusting the curves for age reduced the separation between the curves (Fig. 1).

### HbA_1c_-associated risk in the overall cohort, age groups and individuals with and without DM

**Fig. 2:**
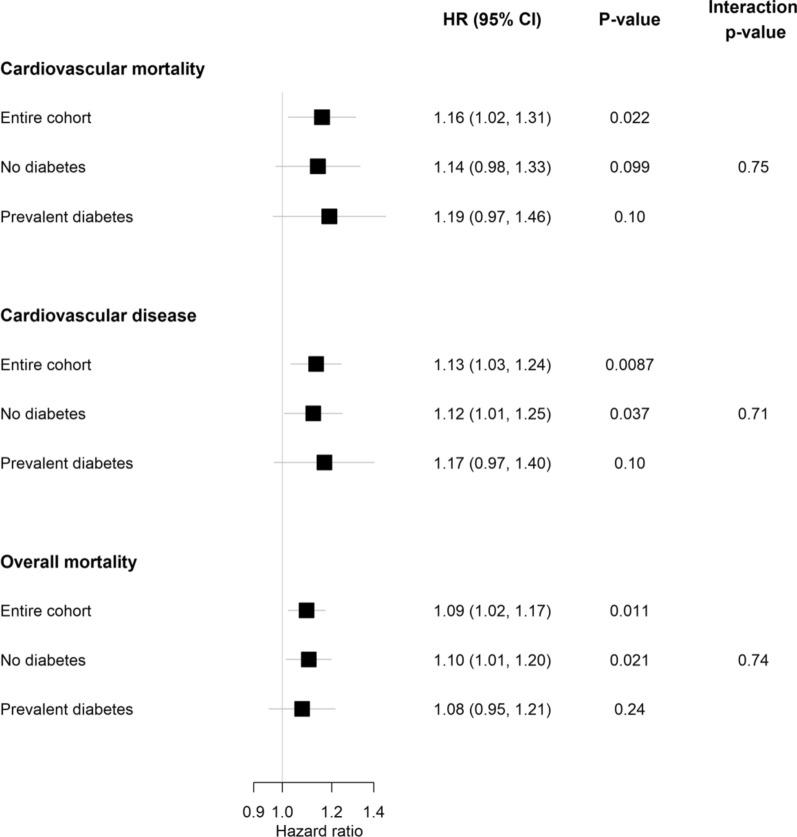
Subgroup analysis comparing the association between HbA_1c_ and time-to-event in individuals with and without DM. HbA_1c_ was used as continuous variable in mmol/mol. HbA_1c_ hazard ratios are presented per 10 mmol/mol increase. The models include an interaction term between HbA_1c_ and the subgroup indicator (DM yes/no). The Cox models for the three endpoints were adjusted for age (time scale), sex and cohort (strata), and CVRFs, smoking status, BMI, systolic blood pressure, DM, DM treatment, and total cholesterol to HDL cholesterol ratio. The p-value for interaction is for an interaction between DM and HbA_1c_

**Fig. 3:**
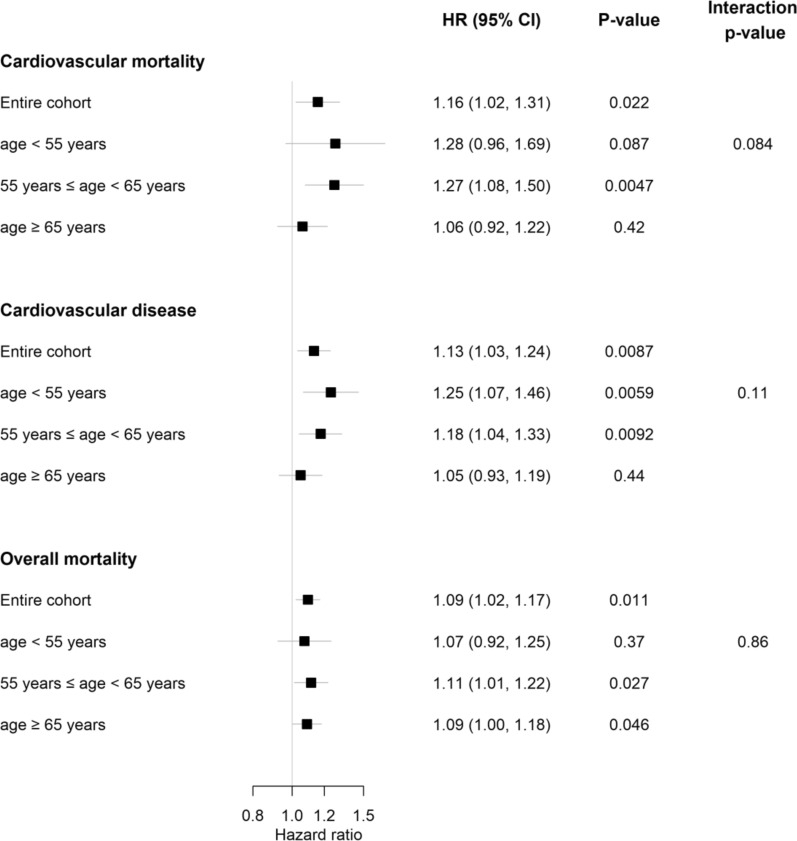
Hazard ratios for HbA_1c_ and outcomes: cardiovascular mortality, cardiovascular disease, and overall mortality, stratified into age groups. HbA_1c_ was used as continuous variable in mmol/mol. Hazard ratios are presented per 10 mmol/mol increase. The Cox models for the three endpoints were adjusted for age (time scale), sex and cohort (strata), and CVRFs, smoking status, BMI, systolic blood pressure, DM, DM treatment, and total cholesterol to HDL cholesterol ratio. The p-value for interaction is for an interaction between age groups and HbA_1c_

The fully adjusted hazard ratios (HRs) shows associations with cardiovascular mortality, CVD incidence, and overall mortality, with respective HR of 1.16 (95% confidence interval (CI): 1.02–1.31, p = 0.02) for cardiovascular mortality, 1.13 (95% CI 1.03–1.24, p = 0.01) for CVD incidence, and 1.09 (95% CI 1.02–1.17, p = 0.01) for overall mortality per 10 mmol/mol increase in HbA_1c_ (Figs. 2 and 3, Additional file 1: Table S4).

In individuals with DM, we observed a HR of 1.19 (95% CI 0.97‒1.46; p = 0.1) for cardiovascular mortality, 1.17 (95% CI 0.97‒1.40; p = 0.1) for CVD incidence, and 1.08 (95% CI 0.95‒1.21; p = 0.24) for overall mortality. In participants without DM, the respective HRs for cardiovascular mortality, CVD incidence, and overall mortality were 1.14 (95% CI 0.98‒1.33; p = 0.1), 1.12 (95% CI 1.01‒1.25; p = 0.04), and 1.10 (95% CI 1.01‒1.20; p = 0.02) (Fig. 2).

Following stratification according to age groups, the association between HbA_1c_ and risk of cardiovascular disease incidence was pronounced in individuals aged < 55 years (HR: 1.25, 95% CI 1.07 –1.46, p = 0.006) but diminished with increasing age. In this context, the results for the cohort with subjects older ≥ 55 years and < 65 years were (HR: 1.18, 95% CI 1.04–1.33; p = 0.01) and not present in individuals older 65 years (HR: 1.05, 95% CI 0.93–1.19; p = 0.44) for cardiovascular disease (Fig. 3).

### Dose–response relationships

**Fig. 4:**
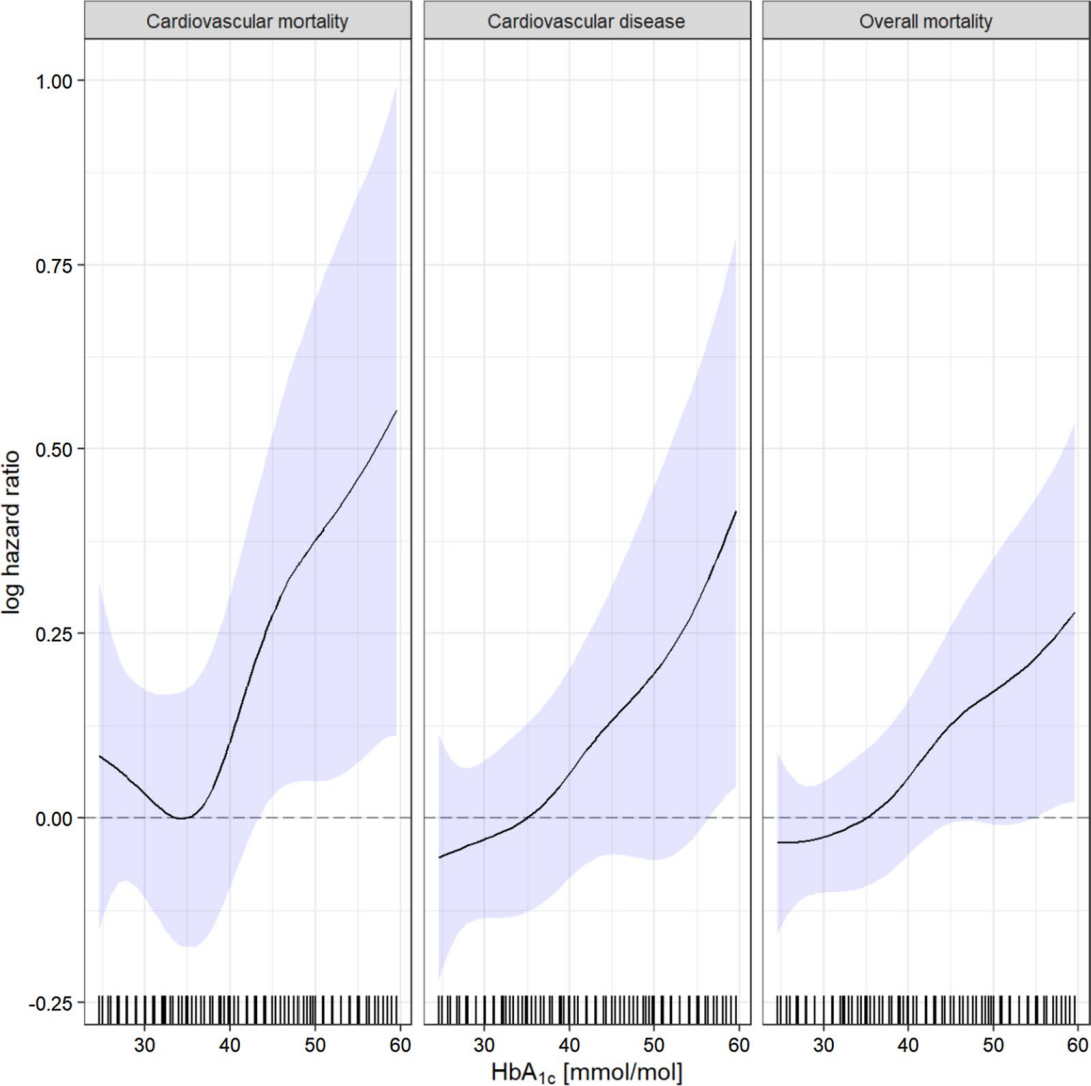
Penalised cubic splines for the association between HbA_1c_ and time-to-event. HbA_1c_ was used as continuous variable and presented in mmol/mol

Modelling the association of HbA_1c_ and time-to-event using cubic splines indicates a slightly curved increasing association for the considered endpoints, with the exception of cardiovascular mortality where the curve decreases until approximately 33 mmol/mol (5.2%) (Fig. 4). The hypothesis of linearity of HbA_1c_ for all 3 endpoints could not be rejected (p = 0.087 for cardiovascular mortality, p = 0.5 for CVD, and p = 0.56 for overall mortality).

### Cut-off value of HbA_1c_ for risk estimation

**Fig. 5:**
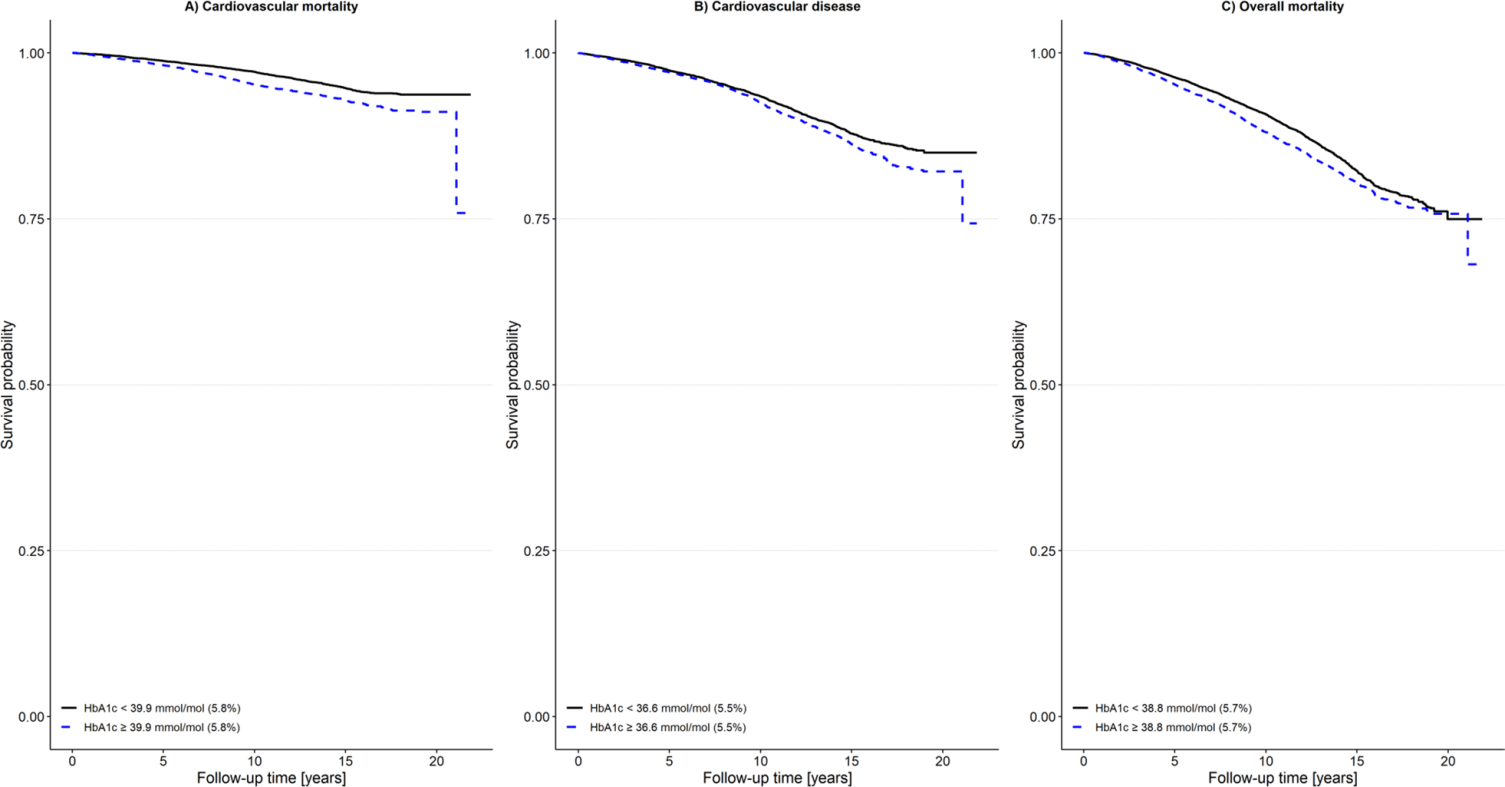
Age adjusted Kaplan–Meier curves for the outcomes **A** cardiovascular mortality, **B** cardiovascular disease, and **C** overall mortality based on the calculated cut-off values

HbA_1c_ cut-off values for cardiovascular mortality, CVD incidence, and overall mortality were calculated, yielding 39.9 mmol/mol (5.8%), 36.6 mmol/mol (5.5%), and 38.8 mmol/mol (5.7%), respectively. These cut-offs indicate an increased risk for the outcome. This is shown by the age-adjusted Kaplan–Meier curves for the three outcomes (Fig. 5). During follow-up, participants that were above the respective cut-off at baseline had a higher risk for each of the outcomes. The unadjusted Kaplan–Meier curves are shown in Additional file 1: Fig. S3.

### Calculation of C-indices

Calculation of C-indices showed no relevant improvement in risk prediction by adding the HbA_1c_ level to the model including the baseline risk factors which were used for adjustment of the Cox regression models for the overall cohort and for individuals without diabetes (Additional file 1: Table S5).

## Discussion

Based on a fairly harmonized large-scale assessment of HbA_1c_ and cardiovascular outcomes, the present study has several main findings. First, HbA_1c_ levels were independently associated with overall-mortality, cardiovascular mortality, and cardiovascular disease. Second, HbA_1c_ levels showed a mostly monotonically increasing association with all three outcomes. Third, the association of HbA_1c_ with cardiovascular disease incidence was strongest in individuals younger than 55 years. Fourth, subgroup analyses based on diabetes status demonstrated that the association between HbA_1c_ and the examined outcomes were significantly associated with CVD incidence and overall mortality in persons without diabetes. Fifth, HbA_1c_ cut-off values were derived to define a threshold above which the risk of the examined outcomes was increased. Finally, despite significant associations of HbA_1c_ with the examined outcomes, 5-year prediction models were not significantly improved by the addition of HbA_1c_ on top of CVRFs.

### HbA_1c_ levels and the risk for cardiovascular outcomes

Although the presence of diabetes is a common risk factor for CVD, a continuous biomarker reflecting this risk factor is not currently used for risk assessment in the general population [6]. In the present prospective population-based study including 36,180 participants from six European countries, we demonstrated a mostly monotonically increasing association for cardiovascular mortality, CVD incidence, and overall mortality with increasing HbA_1c_ levels. The association was mostly monotonic and not J-shaped as reported before [28]. This is in line with a previous meta-analysis of individual level data which overall also did not find a J-shaped association [29]. While this study observed a J-shaped association of HbA_1c_ levels with all cause and cardiovascular mortality in one of the included studies [29] this seemed to be due to confounders like ethnicity, alcohol consumption, BMI, biomarkers of iron deficiency and liver function since the association of very low HbA_1c_ levels with mortality outcomes lost statistical significance after adjusting for these confounders [29].

In this context, the current results with cut-off analysis adjusted for age provided thresholds of HbA_1c_ levels being associated with cardiovascular disease, cardiovascular mortality and overall mortality. These results underline the importance of HbA_1c_ levels below the threshold to diagnose diabetes and to identify individuals with an increased risk. Therefore, our findings underline previous statements from the American Diabetes Association [30] concerning the measurement of HbA_1c_ for cardiovascular risk assessment as well as the recent European Society of Cardiology 2019 guidelines [6] on adults without a diagnosis of diabetes. In this context, neither guideline defines prediabetes as a self-contained clinical entity although individuals with prediabetes have an increased risk to develop diabetes or cardiovascular disease [6, 30, 31]. The American guidelines underline the fact that prediabetes is associated with cardiovascular risk factors like obesity, arterial hypertension, and dyslipidemia and further that levels in the prediabetes range are associated with the risk to develop diabetes. The increasing risk for developing diabetes with increasing HbA_1c_ levels has been reported before [32]. Furthermore, the American guidelines [30, 31] also support the notion of including HbA_1c_ determination in clinical practice for prevention purposes to reduce future CVD burden. The results of the present study support this implication of using HbA_1c_ levels to identify individuals with an increased risk, e.g., classify them as prediabetic based on a cut-off value of > 38.8 mmol/mol (5.7%), especially since all our calculated cut-offs for subject differentiation were below the reported threshold to diagnose diabetes. The cut-off of > 38.8 mmol/mol (5.7%) is also in line with the threshold proposed by the American Diabetes Association to define prediabetes in individuals without a diagnosis of diabetes [30, 31]. In the present study, HbA_1c_ levels above the cut-off value of 36.6 mmol/mol (5.5%) were associated with cardiovascular diseases. This underlines possible implications for levels below the cut-off value of HbA_1c_ used to diagnose prediabetes (i.e. 5.7%).

A recent study in a smaller study population from Spain could also show the additional benefit of including HbA_1c_ levels for the association with CVD [1]. Extending the importance of HbA_1c_ levels in the clinical practice, additional data pointing out the usage of HbA_1c_ levels for risk stratification in patients with CVD or DM with an insufficient glucose control in patients from Asia should be noted [13, 14, 33, 34].

The recent study by Welsh and colleagues using data from the UK only with a median follow-up of 8.9 years also showed an association between HbA_1c_ levels and cardiovascular outcome [2]. The authors suggested that this risk may be increased due to a higher prevalence of CVRFs in the investigated population [2]. However, this interpretation stands in contrast to findings by a different article which showed that the cohort used in the Welsh et al. study had a lower prevalence of risk factors than the average UK population [35].

### Age-dependent effect of HbA_1c_ and risk for cardiovascular outcome

An important finding was that the association between HbA_1c_ and cardiovascular disease incidence was strongest in individuals aged under 55 years. This could imply that young individuals with elevated HbA_1c_ levels may carry an additional risk for developing relevant cardiovascular disease. A possible causal link between HbA_1c_ levels and an increased risk of coronary artery disease was proposed in Mendelian randomization studies [20, 21]. Although the analysis as presented was adjusted for the CVRFs the literature reports that these risk factors might have a different impact on the development of a cardiovascular outcome depending on the age of the individual [36]. Further, older individuals may be less affected by CVRFs since these were present without the individual developing an event prior to the baseline visit.

### HbA_1c_ measurement and its implication regarding the defined outcome

In this large European general population sample the association of HbA_1c_ with the risk of cardiovascular mortality, cardiovascular disease incidence, and overall mortality was mostly monotonic, considering the broad range of HbA_1c_ levels encountered in such large population-based studies [2, 9, 15, 28, 37]. Considering this association, the recent definition of prediabetes [38], and the potentially higher risk of developing diabetes or cardiovascular disease, additional clinical evaluation might be warranted in individuals with a higher risk profile. In our study, CVD incidence was associated with HbA_1c_ levels in participants without diabetes. In addition, the calculated cut-offs showing an elevated risk for overall mortality in individuals with > 38.8 mmol/mol (5.7%) highlights the importance of elevated HbA_1c_ levels and might indicate an increased risk for cardiovascular outcomes and overall mortality. In the context of the current findings, previous studies reported a poor outcome for patients with coronary artery disease, atrial fibrillation or individuals with DM and either cardiovascular risk factors or coronary artery disease [39–41]. However, it is not clarified if these findings are related to the rapid lowering of HbA_1c_ with multiple drugs [41] or due to its proinflammatory, prothrombotic and proatherogenic effects reported in the setting of strict glucose control [42, 43].

It has to be pointed out that measurement of HbA_1c_ levels might be of benefit for identifying individuals with a high risk of developing DM and/or CVD, especially in the presence of risk factors such as obesity, dyslipidemia and/or arterial hypertension. In this context the American Diabetes Association recommends to measure HbA_1c_ levels in asymptomatic young adults [31].

### Risk prediction models including HbA_1c_ levels

Inclusion of HbA_1c_ levels into assessment of risk prediction regarding the defined outcomes did not show an improvement as reflected by C-indices. This is inline with previous reported results [2, 28].

### Strengths and limitations

The present study has several strengths and limitations. An important strength is the considerable size of the dataset, with harmonized data from well-defined European population-based cohort studies with a long follow-up time.

Despite the well-defined dataset, we identified 717 individuals with HbA_1c_ levels above 48 mmol/mol/6.5% that had not been diagnosed with diabetes. As the omission of such a sizable group may introduce considerable errors into our findings, we decided to classify individuals with HbA_1c_ levels above 48 mmol/mol/6.5% and without the diagnosis of diabetes as subjects with prevalent diabetes. An additional limitation is the heterogeneity of data. Several cohort studies whose data we used commenced in the 1980s and 1990s, when treatment options and guidelines differed substantially from today’s. In population-based cohort studies with apparently healthy individuals, selection bias is a common problem.

Haemoglobinopathies, different ethnicities, and certain disease states like bleeding, transfusion, or hemodialysis can interfere with the measurement of HbA_1c_ which may affect our results [38, 44].

Due to the absence of additional measures of dysglycemia like 2-h post load glucose and fasting glucose we could not perform additional analyses to prove and to validate the prognostic impact of HbA_1c_. These parameters were reported to have a prognostic impact and might be better for risk stratification than HbA_1c_ [45, 46].

## Conclusion

The present study employed one of the largest population-based datasets with predominantly harmonized data on HbA_1c_ from several European countries. HbA_1c_ levels were positively associated with an increased risk for cardiovascular mortality, CVD, and overall mortality. There was a mostly monotonically increasing association between HbA_1c_ levels and time-to-event regarding the defined endpoints cardiovascular mortality, cardiovascular disease incidence, and overall mortality, emphasizing the potential use of HbA_1c_ measurement as a biomarker in the general population. Regarding risk stratification, HbA_1c_ levels could be particularly important in subjects with HbA_1c_ levels > 38.8 mmol/mol (5.7%), indicating a potential prediabetic metabolism and potential risk of cardiovascular disease. In addition, the findings might be of particular importance in individuals younger than 55 years who showed a more pronounced association of HbA_1c_ levels and cardiovascular mortality. However, the cost-effectiveness of testing HbA_1c_ levels in an asymptotic general population needs further evaluation.

Further research and external validation in a clinical setting are required to define whether an additional standardized measurement of HbA_1c_ is necessary for cardiovascular risk assessment.

## Supplementary Information

**Additional file 1.** Online supplementary material.

## Data Availability

The data are not available in a public repository. Access to the data is dependent upon ethics approval and restricted by the legislation of the European Union and the countries providing data to the study. Furthermore, approval by the Principal Investigator of each cohort study and the MORGAM/ BiomarCaRE Steering Group is required to release the data. The MORGAM Manual at https://www.thl.fi/publications/morgam/manual/contents.htm gives more information on access to the data.
