# Supplementary material for: Association of glycated hemoglobin A1c levels with cardiovascular outcomes in the general population: results from the BiomarCaRE (Biomarker for Cardiovascular Risk Assessment in Europe) consortium
Source: Cardiovasc Diabetol. 2021 Nov 15;20:223. doi: 10.1186/s12933-021-01413-4 (PMC8594211; doi:10.1186/s12933-021-01413-4)
Supplement: Supplementary file 1 — Additional file 1. Online supplementary material. [file 12933_2021_1413_MOESM1_ESM.docx]

**ADDITIONAL FILES**

**TITLE PAGE**

**Association of glycated hemoglobin A_1c_ levels with cardiovascular outcomes in the general population: Results from the BiomarCaRE (Biomarker for Cardiovascular Risk Assessment in Europe) consortium**

Christoph Sinning^a,b^*^■,^ Nataliya Makarova^a,b^*, Henry Völzke^c,d^, Renate B. Schnabel^a,b^, Francisco Ojeda^a^, Marcus Dörr^d,e^, Stephan B. Felix^d,e^, Wolfgang Koenig^f,g^, Annette Peters^h,t^, Wolfgang Rathmann^i^, Ben Schöttker^j,k^, Hermann Brenner^j,k,^, Giovanni Veronesi^l^, Giancarlo Cesana^m^, Paolo Brambilla^m^, Tarja Palosaari^n^, Kari Kuulasmaa^n^, Inger Njølstad^o^, Ellisiv Bøgeberg Mathiesen^p,q^, Tom Wilsgaard^o^, Stefan Blankenberg^a,b^, Stefan Söderberg^r+^, Marco M. Ferrario^l+^, Barbara Thorand^h,s+^

^a^ University Heart & Vascular Center Hamburg, Department of Cardiology, Hamburg, Germany

^b^ German Center for Cardiovascular Research (DZHK), Partner Site Hamburg/Kiel/Lübeck, Hamburg, Germany

^c^ University Medicine Greifswald, Institute for Community Medicine, Department of Study of Health in Pomerania/Clinical-Epidemiological Research, Greifswald, Germany

^d^ German Center for Cardiovascular Research (DZHK), Partner site Greifswald, Greifswald, Germany

^e^ University of Medicine Greifswald, Department of Internal Medicine B, Greifswald, Germany;

^f^ German Heart Center Munich, Technical University, Munich, Germany; German Center for Cardiovascular Research (DZHK), Partner Site Munich Heart Alliance, Munich, Germany

^g^ Institute of Epidemiology and Medical Biometry, University of Ulm, Ulm, Germany

^h^ Helmholtz Zentrum München, German Research Center for Environmental Health, Institute of Epidemiology, Neuherberg, Germany

^i^ German Diabetes Center Düsseldorf, Institute of Biometrics and Epidemiology, Düsseldorf, Germany

^j^ German Cancer Research Center, Division of Clinical Epidemiology and Ageing Research, Heidelberg, Germany

^k^ University of Heidelberg, Network Aging Research, Heidelberg, Germany

^l^ EPIMED Research Center, Department of Medicine and Surgery, University of Insubria at Varese, Varese, Italy

^m^ Department of Medicine and Surgery, University of Milano-Bicocca, Milan, Italy

^n^ National Institute for Health and Welfare, Helsinki, Finland

^o^ UiT The Arctic University of Norway, Department of Community Medicine, Tromsø, Norway

^p^ UiT The Arctic University of Norway, Brain and Circulation Research Group, Tromsø, Norway

^q^ University Hospital of North Norway, Neurological Department, Tromsø, Norway

^r^ Umeå University, Department of Public Health and Clinical Medicine, Umeå, Sweden

^s^ German Center for Diabetes Research (DZD), München-Neuherberg, Germany

* Co-shared first

+ Co-shared last

**Corresponding author**

Christoph Sinning, MD

University Heart & Vascular Center Hamburg

Department of Cardiology

Martinistr. 52

20246 Hamburg

Germany

Tel: +4915222817675

E-Mail: [c.sinning@uke.de](mailto:c.sinning@uke.de)

**Supplement to the statistical part of the manuscript:**

For each endpoint C-indices were computed for 1) base Cox model, and 2) base + HbA_1c_ Cox model. The base model included the variables age (time scale), sex and cohort (strata), and CVRFs, smoking status (daily smoker yes/no), BMI, systolic blood pressure, DM (yes/no), total cholesterol to HDL cholesterol ratio and treatment of DM. The base + HbA_1c_ model included continuous HbA_1c_ in mmol/mol in addition to all base model variables. 10-fold cross-validation was used in the C-indices computation to correct for the overoptimism of computing model performance measures on the same dataset where the model was fitted. The 5 year event probabilities were used to compute the C-indices (any follow-up longer than that was censored). These probabilities were estimated using a Weibull curve fitted over age and adjusted by the linear predictor of the corresponding Cox model. In the Cox models used continuous variables (with the exception of age) were modelled using cubic splines. Confidence intervals and p-values for differences in the C-indices were computed using the methods described in reference [1].

**Table S1**

Overview and description of contributing studies

**BiomarCaRE cohorts**

| **Study/cohort (Reference)** | **Country** | **Study/cohort full name and short description** |
| --- | --- | --- |
| MONICA Brianza Study[2] | Italy | The MONICA-Brianza Cohort Study is a prospective observational study of three cohorts of 25-64 years old residents in Brianza, a highly-industrialised area located between Milan and the Swiss border, Northern Italy. Gender- and ten-year age stratified samples were randomly drawn in 1986, 1990, and 1993, and cardiovascular risk factors were investigated at baseline following the procedures of the WHO MONICA Project. The overall participation rate was 69%. For all individuals whole blood and serum samples were stored in a biobank. Persons with ICD-9 code 250 were considered having history of diabetes. An affirmative answer to any one of the options "diabetes mellitus, treated by diet only", "diabetes mellitus, treated by oral hypoglycemic agents", "diabetes mellitus, treated by insulin" or "diabetes mellitus, no therapy" under the question "previous medical history" was considered as history of diabetes.  The protocol was approved by the Monza Hospital Ethical Committee. Study participants were followed up for first coronary or stroke events, fatal and non-fatal, up to the end of 2008, for a median of 15 years. Further details on the study procedures can be found at <http://epimed.uninsubria.eu> and https://thl.fi/publications/morgam/cohorts/full/italy/ita-bria.htm. |
| MONICA/Kooperative Gesundheits-forschung in der Region Augsburg (KORA)[3] | Germany | The WHO Multinational Monitoring of Trends and Determinants in Cardiovascular Disease (MONICA)/ Cooperative Health Research in the Region of Augsburg (KORA) cohorts comprise all respondents from representative sample surveys from the city of Augsburg and the less urban Landkreis Augsburg and Landkreis Aichach-Friedberg regions in Bavaria, Southern Germany. List of municipalities and population registers were used as sampling frames for the first and the second stage of two-stage sampling, respectively. The second stage of sampling was stratified by sex and ten-year age group. The Survey 3 (S3) baseline examination (1994-1995) was carried out as part of the WHO MONICA project and consists of 4856 men and women aged 25-74 years with a response rate of 75%. The Survey 4 (S4) baseline examination was carried out in 1999-2001 and includes 4261 participants (response rate: 66%). The S4 study and morbidity and mortality follow-ups were conducted within the frame of KORA. The BiomarCaRE project includes n=4692 and n=4221 participants from S3 and S4, respectively.  Within the framework of the KORA Studies, follow-up questionnaires were sent to each former participant in 1997-1998, in 2002-2003, and in 2008-2009 to obtain information on the occurrence of chronic diseases and risk factors. Mortality follow-up was performed as follows: If a person did not return the follow-up questionnaire, the person's vital status was ascertained through the population registries inside and outside the study area.  Coronary events were identified through the MONICA/KORA Augsburg coronary event registry [4]. Coronary deaths were validated by autopsy reports, death certificates, and chart review from the last treating physician. Self-reported cases of incident stroke were validated by medical records [5]. The diagnostic classification of history of diabetes was done using the general practitioner’s notes or hospital discharge letters. When information on the type of diabetes was not available, it was considered to be type 2 if the age of the person was above 35 years. An affirmative answer to the question "Have you ever been told by a doctor that you have diabetes mellitus?" or if the answer was negative but the person was currently taking medication for diabetes.  Further details on the study procedures can be found under https://thl.fi/publications/morgam/cohorts/full/germany/ger-auga.htm and https://www.helmholtz-muenchen.de/en/kora/. |
| Study of Health in Pomerania (SHIP)[6] | Germany | The SHIP study is an established population-based project conducted in Northeast Germany. The study aims to assess prevalence and incidence of common risk factors, subclinical disorders and clinical diseases and to investigate associations and interactions among them using comprehensive medical assessments. The first SHIP cohort was recruited between 1997 and 2001 and included 4308 individuals at baseline (SHIP-0, 20-70 years, response 68.8%), 3300 after five years (SHIP-1) and 2333 after 11 years (SHIP-2). In parallel to SHIP-2, baseline examinations of a second, independent cohort (SHIP-TREND) were conducted in 4420 participants (20-79 years, response 50.3%). For the current analysis only SHIP-0 was used. SHIP is one of the population-based projects with very comprehensive examinations including interviews, cardio-metabolic ultrasound exams, cardiopulmonary exercise tests and whole-body magnetic resonance imaging in a general population setting. History of diabetes was self-reported or based on relevant information for diabetes derived from the SHIP-database with level of HbA_1c_ > 6.5%, serum glucose > 11.1 mmol/L, or treatment of the subject with antidiabetic medication). In addition to the examination follow-ups, information on fatal and non-fatal disease is collected on a regular basis. Mortality follow-ups are conducted semi-annually by record linkage with data bases of the regional population registry. Causes of death are defined from the official death documents provided by regional health authorities. Active follow-ups for non-fatal diseases are performed biannually and by interviews during follow-up examinations every five years. Self-reported information is validated by GP’s and using databases of the regional Association of SHI Physicians.  Data from the SHIP study were directly transferred to the University Heart Center Hamburg in accordance with a project specific data transfer agreement.  <https://thl.fi/publications/morgam/cohorts/full/germany/ger-grea.htm> and http://www.medizin.uni-greifswald.de/cm/fv/ship.html. |
| ESTHER Study[7, 8] | Germany | ESTHER is a large-scale population-based cohort study, which was initiated to study potential new approaches for the prevention and early detection of chronic diseases. From June 2000 to December 2002, more than 400 general practitioners recruited 9953 participants, born between 1925 and 1952, during a routine health check-up in Saarland, a state located in the south west of Germany. More than 98% of the participants were of German nationality. All members of compulsory health insurances in Germany (90% of the German population) aged 35 years or older are entitled to have these health-screening examinations focusing on early detection of cardiovascular and renal diseases and diabetes mellitus every 2 years. All participants gave written informed consent. The ESTHER study was approved by the Ethics Committees of the Medical Faculty, University of Heidelberg and of the Medical Association of Saarland and is conducted in accordance with the Declaration of Helsinki. All participants completed a standardized questionnaire regarding sociodemographic and lifestyle factors, and their medical history. Special emphasis was made to record lifetime history of lifestyle factors like smoking, alcohol consumption, physical activity or body weight. History of diabetes was defined as known and newly diagnosed diabetes, which has been documented in the health check-up report by primary physicians. In addition, anti-diabetic drug use was reported by the general practitioners. An affirmative answer to the question “Did a physician ever diagnose diabetes?” is also considered as history of diabetes.  <https://thl.fi/morgam/a/publications/cohorts/full/germany/ger-esra.htm> and http://esther.dkfz.org/esther/. |
| The Northern Sweden MONICA Study[9] | Sweden | **The Northern Sweden MONICA Study:** The Northern Sweden MONICA study covered the two northernmost counties of Sweden, i.e. Norrbotten and Västerbotten with altogether 510,000 inhabitants. Population surveys were performed in 1986, 1990, 1994, 1999, 2004 and 2009, with altogether 10,517 unique participants [10]. In the first two surveys, 2,000 persons aged 25 to 64 years were randomly selected, and in the last three surveys, the upper age limit was extended to 74 years and 2,500 individuals were invited. A stratified randomized selection procedure by age and sex (250 persons in each sex/10-year age stratum) was used. The participation rate was 69-81%. Detailed analyses of non-participants have been performed. All participants donated blood to the Northern Sweden Biobank in Umeå, and in 2012 samples from the surveys in 1994 and 2009 were thawed and HbA_1c_ was measured. An affirmative answer to the question "Do you have diabetes?" was considered as history of type 1 or type 2 diabetes. If diabetes had been diagnosed before baseline, this was considered indicative of history of diabetes  Incident cardiovascular events (myocardial infarction and stroke) occurring in the region between 1985 and 2010 and below the age of 75 were collected and validated according to MONICA criteria by two event registers whose accuracy and validity have been tested against national registers. Follow-up is available for all cohorts until December 2011 for mortality and non- fatal coronary, stroke, chronic heart failure, atrial fibrillation, cancer and diabetes events. Coronary and stroke events below the age of 75 validated applying the MONICA diagnostic criteria, and diabetes according to careful case review.[11] <http://www.thl.fi/publications/morgam/cohorts/full/sweden/swe-nswa.htm> |
| The Tromsø Study[12] | Norway | The Tromsø Study was initiated in 1974 in an attempt to help combat the high mortality of cardiovascular diseases in Norway that was particularly pronounced among middle-aged men. In the mid-1970s, Norwegian men had a 20% risk of dying of myocardial infarction (MI) before the age of 75 years. The Tromsø Study consists of seven surveys (referred to as Tromsø 1–7) that have been conducted in the municipality of Tromsø from 1974 to 2016. The BiomarCaRE project includes participants from Tromsø 3 and 4. HbA_1c_ was measured only among those of Tromsø 4 who attended the second visit with more extensive examinations. Therefore, only this subcohort was included in the current analysis. In this group, everyone in age group 55-74 was invited, as well as 5-10% from age groups 25-54 and 75-85.  An affirmative answer to the question "Do you have, or have you had diabetes:" was considered as self-reported history of diabetes. Possible cases of diabetes were identified through linkage of the Tromsø Study participant list to diabetes related discharge diagnoses in the digital patient records at the only local hospital (ICD- 9 codes 250, 357.2, 362.0, 583.8, 648.0, 648.8, 790.2). Manual and electronic searches for notes on diabetes were also done as a part of our adjudication process for cardiovascular diseases. Cases were classified as having diabetes if they had non-fasting glucose ≥ 11.1 mmol/L, fasting glucose > 7.0 mmol/L, 2 h glucose load ≥ 11.1 mmol/L or HbA1c ≥ 7.0% and/or recorded regular use of insulin or oral anti-diabetic drugs. The diagnosis of diabetes was based on information from the medical records.  All participants are being followed-up with regard to mortality and disease incidence. Further details on the study procedures can be found at <https://thl.fi/morgam/a/publications/cohorts/full/norway/nor-tro.htm> and <https://en.uit.no/prosjekter/prosjekt?p_document_id=80172>. |

**Table S2. *Baseline characteristics shown for each study cohort separately***

|  | **All (N=36180)** | **Brianza (N=3155)** | **ESTHER (N=9767)** | **MONICA/KORA (N=8641)** | **Northern Sweden (N=3045)** | **SHIP (N=4402)** | **Tromso (N=7170)** |
| --- | --- | --- | --- | --- | --- | --- | --- |
| **Baseline characteristics** |  |  |  |  |  |  |  |
| Survey year | 1987-2012 NAs:0 | 1987-1994 NAs:0 | 2000-2003 NAs:0 | 1994-2001 NAs:0 | 1994-2009 NAs:0 | 2008-2012 NAs:0 | 1994-1995 NAs:0 |
| Examination age (years) | 57.4 (47.0, 65.1) NAs:0 | 46.7 (36.9, 56.1) NAs:0 | 62.6 (57.1, 67.2) NAs:0 | 50.2 (37.6, 61.7) NAs:0 | 50.6 (38.3, 61.9) NAs:0 | 53.0 (40.0, 64.0) NAs:0 | 60.4 (54.3, 67.4) NAs:0 |
| Male (%) | 17069 (47.2) NAs:0 | 1564 (49.6) NAs:0 | 4404 (45.1) NAs:0 | 4334 (50.2) NAs:0 | 1541 (50.6) NAs:0 | 2133 (48.5) NAs:0 | 3093 (43.1) NAs:0 |
| BMI (kg/m²) | 26.4 (23.8, 29.4) NAs:144 | 25.0 (22.6, 28.0) NAs:36 | 27.3 (24.8, 30.1) NAs:13 | 26.5 (23.9, 29.6) NAs:60 | 25.8 (23.3, 28.7) NAs:17 | 27.5 (24.5, 31.0) NAs:4 | 25.5 (23.2, 28.2) NAs:14 |
| Daily smoker (%) | 8243 (27.7) NAs:6464 | 910 (28.9) NAs:2 | 1438 (43.0) NAs:6425 | 1957 (22.7) NAs:4 | 500 (16.5) NAs:17 | 1179 (26.9) NAs:13 | 2259 (31.5) NAs:3 |
| Hypertension (%) | 17084 (47.5) NAs:229 | 1046 (33.4) NAs:27 | 5226 (54.5) NAs:173 | 3429 (39.8) NAs:16 | 1098 (36.1) NAs:4 | 2100 (47.8) NAs:9 | 4185 (58.4) NAs:0 |
| Systolic BP (mmHg) | 133.5 (120.0, 149.0) NAs:286 | 126.0 (114.0, 140.0) NAs:30 | 140.0 (130.0, 150.0) NAs:225 | 129.5 (118.0, 144.0) NAs:16 | 126.0 (115.0, 140.0) NAs:4 | 127.0 (114.5, 139.5) NAs:11 | 142.5 (129.5, 159.5) NAs:0 |
| Diastolic BP (mmHg) | 80.0 (74.0, 90.0) NAs:290 | 81.0 (74.0, 89.0) NAs:31 | 80.0 (80.0, 90.0) NAs:228 | 80.0 (73.0, 88.0) NAs:16 | 79.0 (72.0, 87.0) NAs:4 | 76.5 (70.0, 83.5) NAs:11 | 83.0 (75.0, 92.5) NAs:0 |
| Antihypertensive (%) | 7827 (21.7) NAs:177 | 358 (11.4) NAs:21 | 3371 (34.7) NAs:43 | 1242 (14.4) NAs:11 | 442 (14.8) NAs:67 | 1527 (34.7) NAs:4 | 887 (12.4) NAs:31 |
| Diabetes (%) | 3684 (10.2) NAs:0 | 235 (7.4) NAs:0 | 1842 (18.9) NAs:0 | 530 (6.1) NAs:0 | 243 (8.0) NAs:0 | 550 (12.5) NAs:0 | 284 (4.0) NAs:0 |
| Diabetes treatment: none (%) | 33834 (95.1) NAs:608 | 3065 (98.8) NAs:54 | 8580 (92.2) NAs:465 | 8276 (95.8) NAs:5 | 2926 (96.3) NAs:8 | 4011 (91.2) NAs:4 | 6976 (98.3) NAs:72 |
| Diabetes treatment: insulin (%) | 598 (1.7) NAs:608 | 2 (0.1) NAs:54 | 241 (2.6) NAs:465 | 96 (1.1) NAs:5 | 50 (1.6) NAs:8 | 157 (3.6) NAs:4 | 52 (0.7) NAs:72 |
| Diabetes treatment: tablets, but no insulin (%) | 1007 (2.8) NAs:608 | 26 (0.8) NAs:54 | 481 (5.2) NAs:465 | 187 (2.2) NAs:5 | 39 (1.3) NAs:8 | 204 (4.6) NAs:4 | 70 (1.0) NAs:72 |
| Diabetes treatment: dietary (%) | 133 (0.4) NAs:608 | 8 (0.3) NAs:54 | 0 (0) NAs:465 | 77 (0.9) NAs:5 | 22 (0.7) NAs:8 | 26 (0.6) NAs:4 | 0 (0) NAs:72 |
| Family history of CHD (%) | 4716 (18.6) NAs:10825 | 553 (17.6) NAs:14 | 1216 (12.6) NAs:98 | 848 (19.1) NAs:4207 | 317 (13.1) NAs:627 | - NAs:4402 | 1782 (31.3) NAs:1477 |
| Prev. MI or stroke (%) | 2132 (6.0) NAs:404 | 58 (1.8) NAs:6 | 798 (8.5) NAs:377 | 283 (3.3) NAs:10 | 179 (5.9) NAs:0 | 215 (4.9) NAs:11 | 599 (8.4) NAs:0 |
| History of MI (%) | 1417 (4.0) NAs:307 | 36 (1.1) NAs:6 | 522 (5.5) NAs:293 | 174 (2.0) NAs:0 | 111 (3.6) NAs:0 | 132 (3.0) NAs:8 | 442 (6.2) NAs:0 |
| Prev. Stroke (%) | 862 (2.4) NAs:339 | 23 (0.7) NAs:6 | 329 (3.5) NAs:316 | 132 (1.5) NAs:10 | 78 (2.6) NAs:0 | 100 (2.3) NAs:7 | 200 (2.8) NAs:0 |
| History of heart failure (%) | 1454 (5.7) NAs:10697 | - NAs:3155 | 980 (10.1) NAs:77 | 331 (4.0) NAs:273 | 21 (0.7) NAs:5 | 122 (2.8) NAs:17 | - NAs:7170 |
| **Endpoints** |  |  |  |  |  |  |  |
| Cardiovascular mortality (%) | 1392 (3.9) NAs:83 | 58 (1.8) NAs:0 | 315 (3.2) NAs:0 | 260 (3.0) NAs:0 | 95 (3.1) NAs:0 | 24 (0.6) NAs:83 | 640 (8.9) NAs:0 |
| Cardiovascular disease (%) | 2339 (8.2) NAs:7686 | 202 (6.5) NAs:64 | 182 (2.2) NAs:1459 | 409 (5.3) NAs:983 | 216 (7.5) NAs:179 | - NAs:4402 | 1330 (20.2) NAs:599 |
| Overall mortality (%) | 4601 (12.7) NAs:69 | 309 (9.8) NAs:0 | 1137 (11.6) NAs:1 | 852 (9.9) NAs:0 | 278 (9.1) NAs:0 | 135 (3.1) NAs:68 | 1890 (26.4) NAs:0 |
| **Biomarkers** |  |  |  |  |  |  |  |
| HbA_1c_ (mmol/mol) | 36.6 (32.2, 39.9) NAs:0 | 35.5 (30.1, 41.0) NAs:0 | 37.7 (34.4, 42.1) NAs:0 | 34.4 (31.1, 38.8) NAs:0 | 38.0 (35.0, 42.0) NAs:0 | 34.4 (30.1, 38.8) NAs:0 | 35.5 (32.2, 37.7) NAs:0 |
| HbA_1c_ (%) | 5.5 (5.1, 5.8) NAs:0 | 5.4 (4.9, 5.9) NAs:0 | 5.6 (5.3, 6.0) NAs:0 | 5.3 (5.0, 5.7) NAs:0 | 5.6 (5.4, 6.0) NAs:0 | 5.3 (4.9, 5.7) NAs:0 | 5.4 (5.1, 5.6) NAs:0 |
| Total cholesterol (mmol/L) | 5.9 (5.0, 6.7) NAs:60 | 5.5 (4.8, 6.3) NAs:10 | 5.7 (4.8, 6.5) NAs:30 | 5.8 (5.1, 6.6) NAs:2 | 5.8 (5.0, 6.6) NAs:4 | 5.4 (4.6, 6.1) NAs:1 | 6.6 (5.8, 7.5) NAs:13 |
| HDL cholesterol (mmol/L) | 1.4 (1.2, 1.7) NAs:3773 | 1.4 (1.2, 1.7) NAs:9 | 1.3 (1.1, 1.6) NAs:3705 | 1.4 (1.1, 1.7) NAs:12 | 1.4 (1.2, 1.7) NAs:25 | 1.4 (1.2, 1.7) NAs:1 | 1.5 (1.2, 1.8) NAs:21 |

Baseline characteristics are presented as absolute and relative frequencies for categorical variables, and quartiles (medians with 25th and 75th percentiles) for continuous variables as well as range in years for years of baseline examinations. BMI, body mass index; BP, blood pressure; CHD, coronary heart disease; HDL, high density lipoprotein; LDL, low density lipoprotein; MI, myocardial infarction.

**Table S3** Follow-up time of the different studies in years

| **Cohort study** | **Cardiovascular mortality** | **Cardiovascular disease** | **Overall mortality** |
| --- | --- | --- | --- |
| **Brianza** | 15.1 (14.6, 19.2) | 15.0 (14.5, 19.2) | 18.5 (14.6, 19.2) |
| **ESTHER** | 9.5 (9.0, 9.9) | 7.9 (5.5, 8.2) | 9.6 (9.1, 10.0) |
| **MONICA/KORA** | 9.7 (8.8, 14.0) | 9.4 (8.6, 14.1) | 13.0 (8.9, 14.1) |
| **Northern Sweden** | 3.0 (2.8, 17.9) | 5.9 (2.8, 17.9) | 7.1 (2.8, 17.9) |
| **SHIP** | 2.3 (1.4, 3.2) |  | 2.3 (1.4, 3.2) |
| **Tromso** | 15.8 (15.6, 16.1) | 15.8 (15.6, 16.0) | 15.9 (15.7, 16.1) |

Quartiles of follow-up time (in years) were estimated by the Kaplan-Meier potential follow-up estimator.

**Table S4** Cox proportional hazards models for HbA_1c_ as continuous variable. Hazard ratios for HbA_1c_ are presented per 1 mmol/mol increase.

| 1. Cardiovascular mortality | | | |
| --- | --- | --- | --- |
|  | **Beta (95% CI)** | **HR (95% CI)** | **p-value** |
| HbA_1c_, mmol/mol | 0.01 (0.00, 0.03) | 1.01 (1.00, 1.03) | 0.022 |
| BMI, kg/m² | -0.17 (-0.25, -0.08) |  | <0.001 |
| BMI^2^, (kg/m²)^2^ | 0.003 (0.002, 0.004) |  | <0.001 |
| Systolic BP, mmHg | 0.01 (0.01, 0.01) | 1.01 (1.01, 1.01) | <0.001 |
| Total cholesterol/HDL cholesterol | 0.09 (0.05, 0.13) | 1.09 (1.05, 1.13) | <0.001 |
| Diabetes | 0.33 (0.00, 0.66) | 1.39 (1.00, 1.93) | 0.048 |
| Daily smoker | 0.65 (0.51, 0.78) | 1.91 (1.67, 2.19) | <0.001 |
| Diabetes treatment: Insulin | 0.72 (0.31, 1.13) | 2.06 (1.37, 3.10) | <0.001 |
| Diabetes treatment: Tablets, but no insulin | 0.28 (-0.09, 0.66) | 1.33 (0.91, 1.93) | 0.14 |
| Diabetes treatment: dietary | -0.10 (-0.82, 0.61) | 0.90 (0.44, 1.85) | 0.78 |
| N | 27759 |  |  |
| N events | 1106 |  |  |

| 1. Cardiovascular disease | | | |
| --- | --- | --- | --- |
|  | **Beta (95% CI)** | **HR (95% CI)** | **p-value** |
| HbA_1c_, mmol/mol | 0.01 (0.00, 0.02) | 1.01 (1.00, 1.02) | 0.0087 |
| BMI, kg/m² | -0.03 (-0.11, 0.06) |  | 0.54 |
| BMI^2^, (kg/m²)^2^ | 0.001 (-0.001, 0.002) |  | 0.32 |
| Systolic BP, mmHg | 0.01 (0.01, 0.01) | 1.01 (1.01, 1.01) | <0.001 |
| Total cholesterol/HDL cholesterol | 0.13 (0.10, 0.16) | 1.14 (1.11, 1.17) | <0.001 |
| Diabetes | 0.17 (-0.10, 0.44) | 1.19 (0.91, 1.56) | 0.21 |
| Daily smoker | 0.56 (0.46, 0.65) | 1.75 (1.59, 1.92) | <0.001 |
| Diabetes treatment: Insulin | 0.79 (0.42, 1.16) | 2.20 (1.52, 3.20) | <0.001 |
| Diabetes treatment: Tablets, but no insulin | 0.38 (0.06, 0.71) | 1.46 (1.06, 2.03) | 0.021 |
| Diabetes treatment: dietary | 0.34 (-0.22, 0.90) | 1.41 (0.80, 2.47) | 0.23 |
| N | 21474 |  |  |
| N events | 2147 |  |  |

| 1. Overall mortality | | | |
| --- | --- | --- | --- |
|  | **Beta (95% CI)** | **HR (95% CI)** | **p-value** |
| HbA_1c_, mmol/mol | 0.01 (0.00, 0.02) | 1.01 (1.00, 1.02) | 0.011 |
| BMI, kg/m² | -0.21 (-0.26, -0.17) |  | <0.001 |
| BMI^2^, (kg/m²)^2^ | 0.004 (0.003, 0.004) |  | <0.001 |
| Systolic BP, mmHg | 0.01 (0.00, 0.01) | 1.01 (1.00, 1.01) | <0.001 |
| Total cholesterol/HDL cholesterol | 0.01 (-0.01, 0.04) | 1.01 (0.99, 1.04) | 0.20 |
| Diabetes | 0.26 (0.07, 0.45) | 1.29 (1.07, 1.56) | 0.0074 |
| Daily smoker | 0.70 (0.63, 0.77) | 2.01 (1.87, 2.17) | <0.001 |
| Diabetes treatment: Insulin | 0.65 (0.40, 0.89) | 1.91 (1.49, 2.45) | <0.001 |
| Diabetes treatment: Tablets, but no insulin | 0.30 (0.08, 0.52) | 1.34 (1.08, 1.67) | 0.0082 |
| Diabetes treatment: dietary | -0.69 (-1.24, -0.14) | 0.50 (0.29, 0.87) | 0.015 |
| N | 27839 |  |  |
| N events | 3636 |  |  |

HbA_1c_ in mmol/mol was included untransformed into the analysis. HR stands for hazard ratio. 95% CI stands for 95% confidence interval.

**Table S5** Calculated C-indices for the baseline model and the baseline model including untransformed HbA_1c_ levels.

1. Cardiovascular mortality

| Cohort | C-index (95% CI) Base model | C-index (95% CI) Base model + HbA_1c_ | C-index difference (95% CI) | p-value |
| --- | --- | --- | --- | --- |
| Entire cohort | 0.823 (0.803, 0.843) | 0.823 (0.803, 0.843) | 0.000 (-0.002, 0.002) | 0.91 |
| No diabetes | 0.823 (0.801, 0.846) | 0.825 (0.803, 0.847) | 0.002 (0.000, 0.004) | 0.12 |

1. Cardiovascular disease

| Cohort | C-index (95 CI%) Base model | C-index (95 CI%) Base model + HbA1c | C-index difference (95 CI%) | p-value |
| --- | --- | --- | --- | --- |
| Entire cohort | 0.812 (0.797, 0.826) | 0.811 (0.796, 0.826) | -0.001 (-0.002, 0.000) | 0.11 |
| No diabetes | 0.810 (0.794, 0.826) | 0.810 (0.794, 0.826) | -0.001 (-0.001, 0.000) | 0.16 |

1. Overall mortality

| Cohort | C-index (95 CI%) Base model | C-index (95 CI%) Base model + HbA1c | C-index difference (95 CI%) | p-value |
| --- | --- | --- | --- | --- |
| Entire cohort | 0.808 (0.795, 0.821) | 0.808 (0.795, 0.820) | 0.000 (-0.001, 0.000) | 0.30 |
| No diabetes | 0.804 (0.789, 0.818) | 0.803 (0.789, 0.818) | 0.000 (-0.001, 0.001) | 0.74 |

**Figure S1** Distribution of HbA_1c_. Density histogram of HbA_1c_ levels in the entire study population.


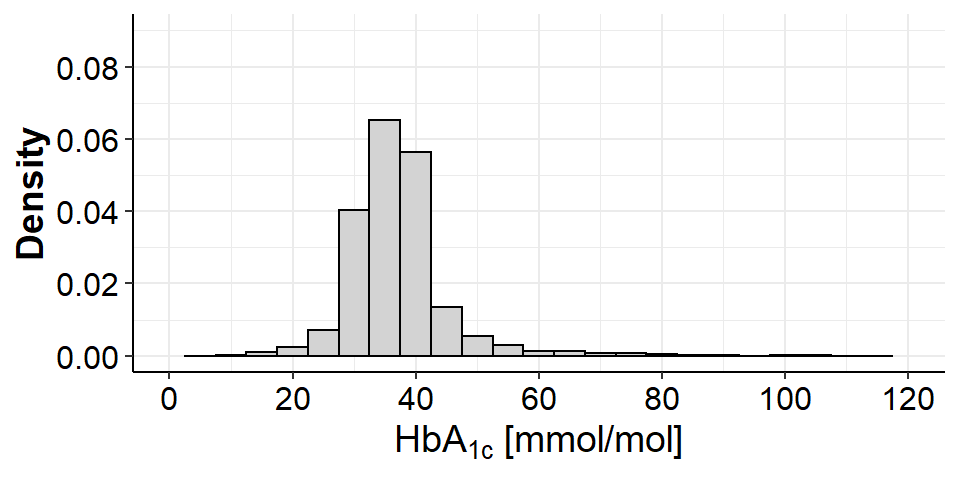


1. in each cohort


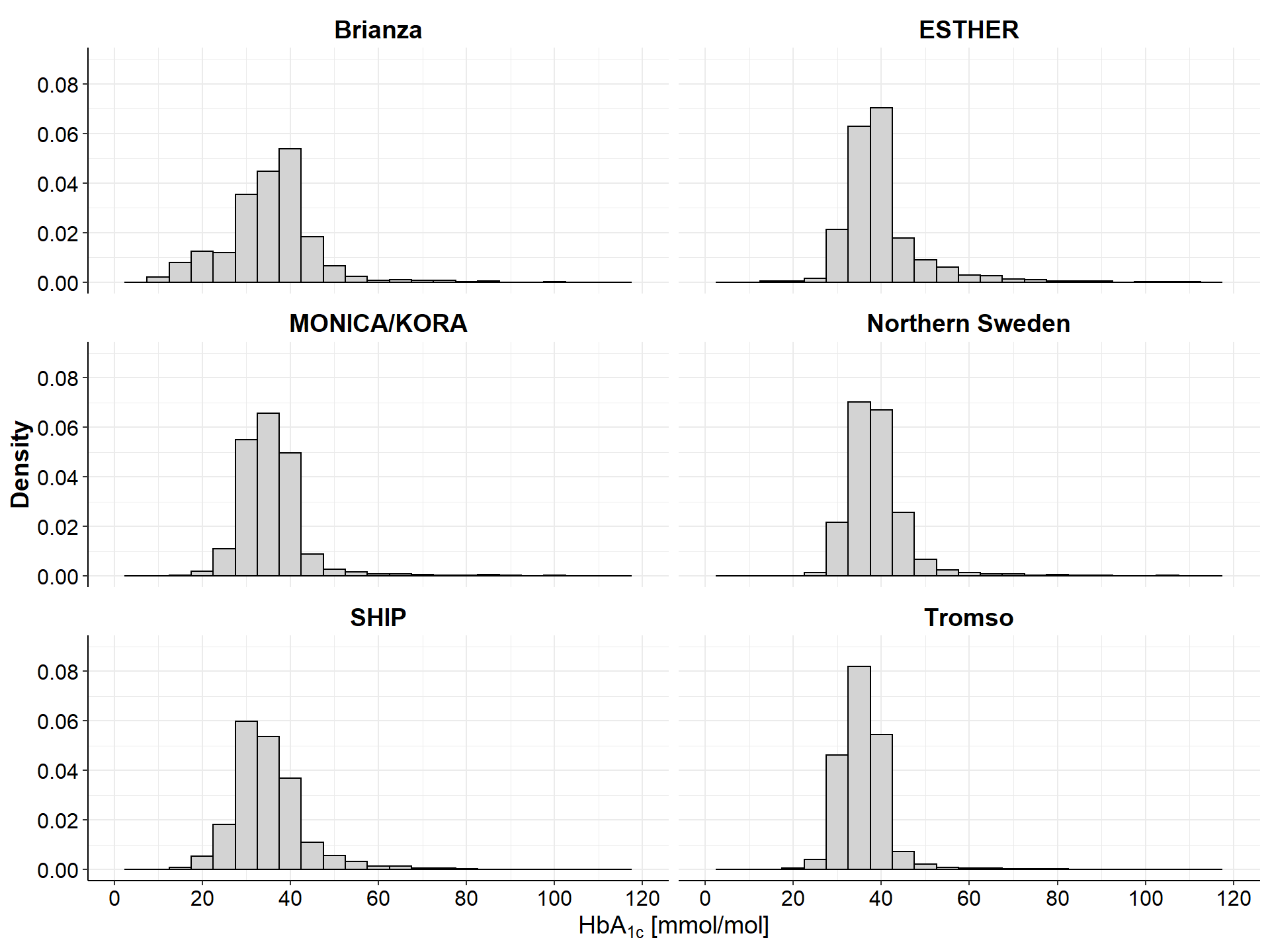


**Figure S2** Unadjusted Kaplan–Meier curves of cardiovascular mortality (A), cardiovascular disease (B), and overall mortality (C) according to thirds of HbA_1c_.


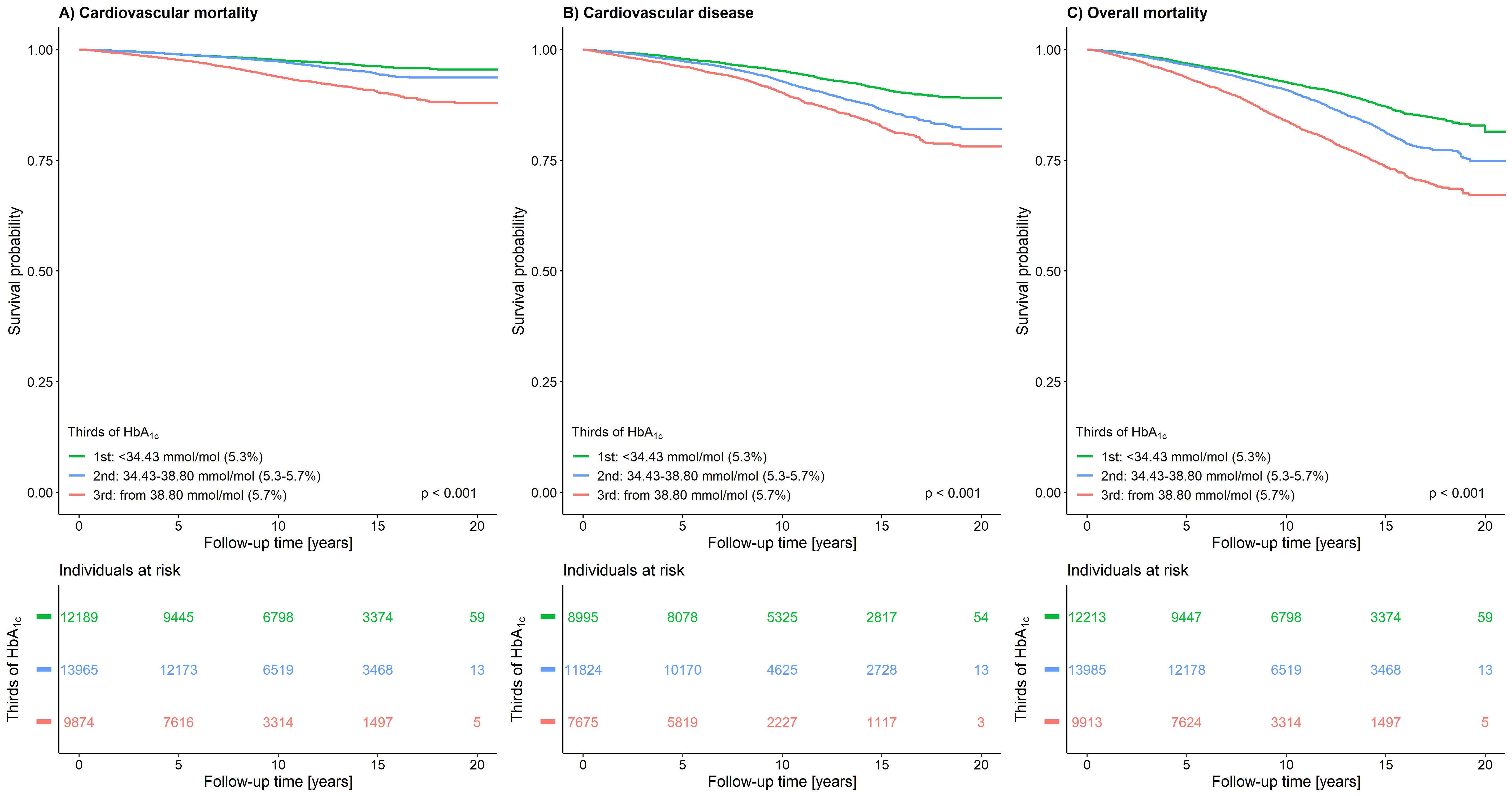


**Figure S3** Unadjusted Kaplan–Meier curves

**
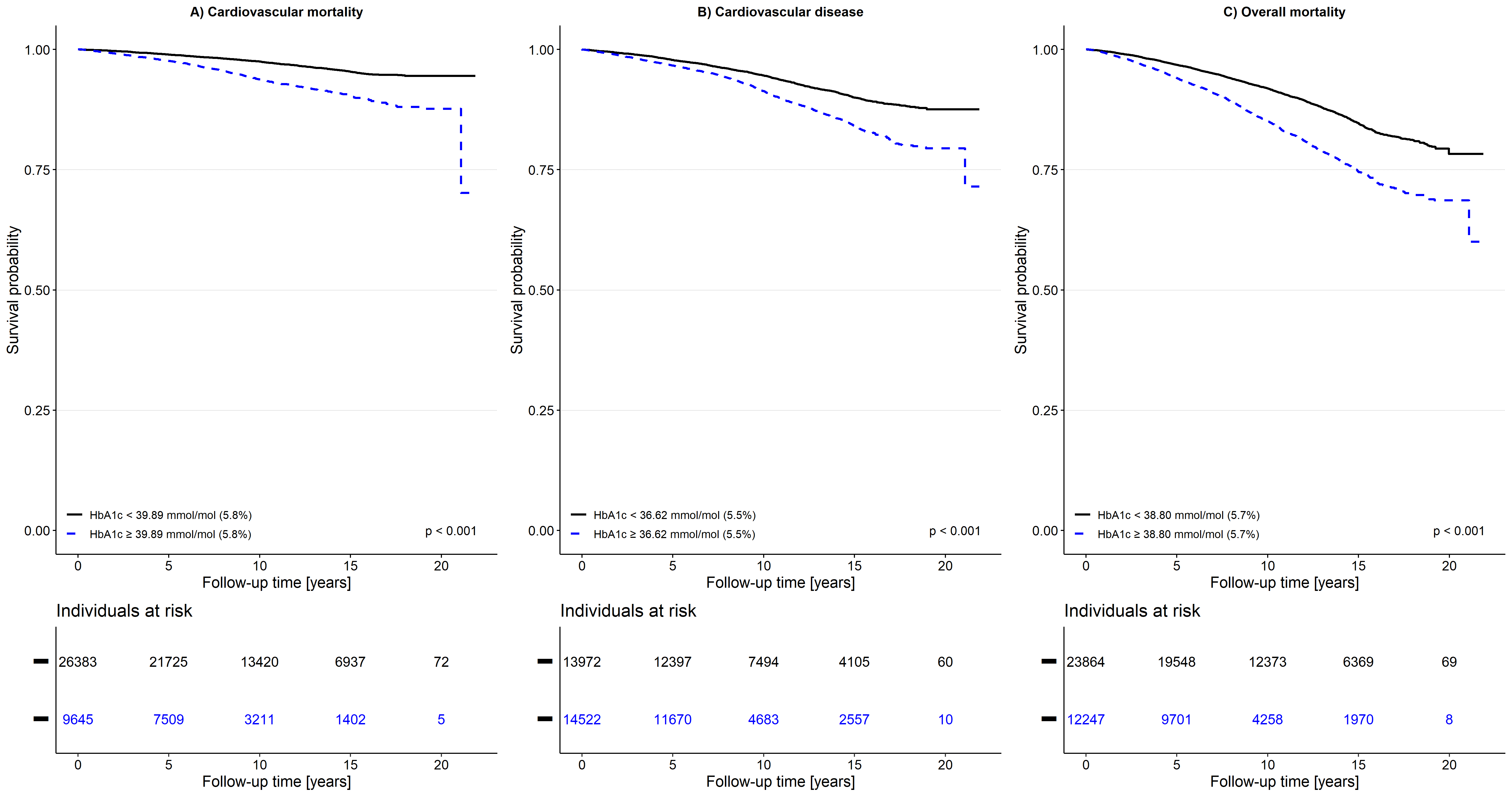
**

**4. References**

1. Antolini LN, B-H.; D'Agostino, R.B.: **Inference on correlated discrimination measures in survival analysis: a nonparametric approach**. *Communications in statistics-Theory and Methods* 2004, **33**(2117-2135).

2. Gianfagna F, Veronesi G, Guasti L, Chambless LE, Brambilla P, Corrao G, Mancia G, Cesana G, Ferrario MM: **Do apolipoproteins improve coronary risk prediction in subjects with metabolic syndrome? Insights from the North Italian Brianza cohort study**. *Atherosclerosis* 2014, **236**(1):175-181.

3. Löwel H, Döring A, Schneider A, Heier M, Thorand B, Meisinger C, Group nftMKS: **The MONICA Augsburg surveys-basis for prospective cohort studies**. *Das Gesundheitswesen* 2005, **67**(S 01):13-18.

4. Holle R, Happich M, Löwel H, Wichmann H-E, Group nftMKS: **KORA-a research platform for population based health research**. *Das Gesundheitswesen* 2005, **67**(S 01):19-25.

5. Löwel H, Lewis M, Hörmann A, Keil U: **Case finding, data quality aspects and comparability of myocardial infarction registers: results of a south German register study**. *Journal of clinical epidemiology* 1991, **44**(3):249-260.

6. Völzke H, Alte D, Schmidt CO, Radke D, Lorbeer R, Friedrich N, Aumann N, Lau K, Piontek M, Born G: **Cohort profile: the study of health in Pomerania**. *International journal of epidemiology* 2010, **40**(2):294-307.

7. Schottker B, Herder C, Rothenbacher D, Roden M, Kolb H, Muller H, Brenner H: **Proinflammatory cytokines, adiponectin, and increased risk of primary cardiovascular events in diabetic patients with or without renal dysfunction: results from the ESTHER study**. *Diabetes Care* 2013, **36**(6):1703-1711.

8. Schottker B, Herder C, Muller H, Brenner H, Rothenbacher D: **Clinical utility of creatinine- and cystatin C-based definition of renal function for risk prediction of primary cardiovascular events in patients with diabetes**. *Diabetes Care* 2012, **35**(4):879-886.

9. Eriksson M, Holmgren L, Janlert U, Jansson JH, Lundblad D, Stegmayr B, Söderberg S, Eliasson M: **Large improvements in major cardiovascular risk factors in the population of northern Sweden: the MONICA study 1986–2009**. *Journal of internal medicine* 2011, **269**(2):219-231.

10. Eriksson M, Holmgren L, Janlert U, Jansson JH, Lundblad D, Stegmayr B, Soderberg S, Eliasson M: **Large improvements in major cardiovascular risk factors in the population of northern Sweden: the MONICA study 1986-2009**. *J Intern Med* 2011, **269**(2):219-231.

11. Rolandsson O, Norberg M, Nystrom L, Soderberg S, Svensson M, Lindahl B, Weinehall L: **How to diagnose and classify diabetes in primary health care: lessons learned from the Diabetes Register in Northern Sweden (DiabNorth)**. *Scand J Prim Health Care* 2012, **30**(2):81-87.

12. Jacobsen BK, Eggen AE, Mathiesen EB, Wilsgaard T, Njølstad I: **Cohort profile: the Tromsø study**. *International journal of epidemiology* 2011, **41**(4):961-967.
